# An imperfect spectrum sensing-based multi-hop clustering routing protocol for cognitive radio sensor networks

**DOI:** 10.1038/s41598-023-31865-5

**Published:** 2023-03-24

**Authors:** Jihong Wang, Chang Liu

**Affiliations:** School of Electrical Engineering, Northeast Electric Power University, Jilin, 132012 China

**Keywords:** Information technology, Electrical and electronic engineering

## Abstract

Multi-hop clustering routing protocols are potential solutions to achieve effective and energy-efficient data delivery in cognitive radio sensor networks (CRSNs). Current clustering routing protocols for CRSNs generally assume perfect spectrum sensing, i.e., false alarm and missed detection are ignored, which may result in collisions with primary users and transmission failure or constrained spectrum usage. To alleviate the impact of imperfect spectrum sensing on network performance, an imperfect spectrum sensing-based multi-hop clustering routing protocol (ISSMCRP) is proposed for CRSNs in this paper. Cluster head (CH) and relay selection criteria are defined based on detection level function of available channels to help select CHs and relays with high spectrum sensing capability. Idle detection accuracy-based intra-cluster and inter-cluster channel selection criterion is proposed to promote successful intra-cluster and inter-cluster data delivery. In addition, control overhead introduced by CHs selection and cluster formation is strictly controlled, and reasonable cluster radii are set to manage the range of control information exchange, so as to reduce the energy consumption caused by control overhead. Simulation results show that compared with the existing clustering routing protocols for CRSNs, ISSMCRP gains obvious advantages in expanding the network lifespan and enhancing the network surveillance capability.

## Introduction

Cognitive radio sensor networks (CRSNs) are intelligent combinations of cognitive radio (CR) technology and wireless sensor networks (WSNs)^1^, in which CRSNs nodes are enabled to opportunistically access the idle spectrum licensed to primary users (PUs) by perceiving the radio spectrum environment^2^. Correspondingly, CRSNs gain obvious advantages in strong universality and high spectral efficiency^3^. However, limited transmission range restricts the effective data delivery from CRSNs nodes to the sink. Clustering routing protocols logically group adjacent nodes into clusters, and multi-hop data delivery is achieved through intra-cluster aggregation and inter-cluster relay, which brings significant performance enhancement for CRSNs^4^. Therefore, clustering routing protocol design for CRSNs has become a hot research topic.

Current clustering routing protocols for CRSNs usually assume perfect spectrum sensing. Although this assumption is beneficial for simplifying protocol design, it does not match the actual perceptual performance of CRSNs nodes. Due to the impact of negative environmental factors, sensing errors such as false alarm and missed detection may occur when a single CRSNs node performs spectrum sensing^5^. For PUs, higher detection probability means less chance of conflict, and it is in favor of normal communications. For CRSNs nodes, lower false alarm probability means a greater chance of leveraging idle licensed spectrum for communication. Thus, guaranteeing high detection probability and low false alarm probability plays a key role in enhancing the system performance^6^. In order to alleviate the negative impact of imperfect spectrum sensing on the network performance, by giving full consideration to spectrum sensing capability of CRSNs nodes, an imperfect spectrum sensing-based multi-hop clustering routing protocol (ISSMCRP) is proposed for CRSNs in this paper. It can provide strong network surveillance capability while extending the network lifespan. The innovations of this paper are summarized as follows:

- Detection level function of available channels is defined to accurately quantify the spectrum sensing capability of CRSNs nodes, based on which cluster head (CH) and next-hop relay selection criteria are proposed to assist in determining high-quality CHs and relays to achieve distributed cluster formation and multi-hop inter-cluster route selection.
- Idle detection accuracy-based intra-cluster and inter-cluster channel selection criterion is proposed to alleviate the impact of missed detection on effective channel selection and promote successful intra-cluster and inter-cluster data delivery.
- Control overhead and the corresponding energy consumption are strictly controlled in ISSMCRP. Reasonable cluster radii are set to manage the range of control information exchange and balance the residual energy among nodes for long network lifetime. Simulation results show that ISSMCRP is superior to the existing clustering routing protocols for CRSNs in extending the network lifetime and enhancing the network surveillance capability.

## Related work

According to cluster formation strategy, current clustering routing protocols for CRSNs are classified into single-hop and multi-hop clustering routing protocols. Single-hop clustering routing protocols require all CRSNs nodes or CHs to reach the sink through single-hop communication. Multi-hop clustering routing protocols can solve the inter-cluster routing problem, and CRSNs nodes which cannot reach the sink directly are enabled to transfer data towards the sink with the assistance of relays. Current clustering routing protocols for CRSNs are reviewed from the above 2 aspects in the following subsections and their comparisons are shown in Table 1. Here, “√” and “×” represent whether the protocol possesses corresponding characteristics.

**Table 1:** Characteristics analysis and comparison of the existing clustering routing protocols for CRSNs.

Protocols	Characteristics analysis			
	Single-hop/Multi-hop	Centralized/Distributed	Uniform/Uneven clustering	Imperfect spectrum sensing
CogLEACH-C^7^	Single-hop	Centralized	Uniform clustering	×
Fuzzy C-means^8^	Single-hop	Centralized	Uniform clustering	×
IMOCRP^9^	Single-hop	Centralized	Uniform clustering	×
TD-IMOCRP^10^	Single-hop	Centralized	Uniform clustering	×
CogLEACH^11^	Single-hop	Distributed	Uniform clustering	×
NSAC^12^	Single-hop	Distributed	Uniform clustering	×
WCM^13^	Single-hop	Distributed	Uniform clustering	×
DSAC^14^	Multi-hop	Distributed	Uniform clustering	×
EACRP^15^	Multi-hop	Distributed	Uniform clustering	×
SACR^16^	Multi-hop	Distributed	Uniform clustering	×
RFMCRP^17^	Multi-hop	Distributed	Uniform clustering	×
EAQ-AODV^18^	Multi-hop	Distributed	Uniform clustering	×
LEAUCH^19^	Multi-hop	Distributed	Uneven clustering	×
PSOEECA^20^	Multi-hop	Distributed	Uneven clustering	×
ESAUC^21^	Multi-hop	Distributed	Uneven clustering	×
ISSMCRP	Multi-hop	Distributed	Uneven clustering	√

### Single-hop clustering routing protocols for CRSNs

Single-hop clustering routing protocols for CRSNs are further divided into centralized and distributed clustering protocols. Centralized clustering protocols such as CogLEACH-C^7^, Fuzzy C-means^8^, IMOCRP^9^ and TD-IMOCRP^10^ require all CRSNs nodes to transmit their residual energy, available channels and other information to the sink. The sink will substitute for normal nodes to select CHs, construct clusters and broadcast the clustering results to the whole network. CogLEACH-C determines the optimal CHs according to the number of available channels, residual energy and the Euclidean distance to the sink. Fuzzy C-means divides the whole network into multiple clusters with the purpose of minimizing the summation of the squared distance between cluster members (CMs) and their CHs. CHs are chosen according to the node residual energy, signal to noise ratio of the report channel with the sink, average path loss with other nodes in the same cluster and path loss with the sink. IMOCRP automatically determines CHs and the optimal number of clusters by leveraging ions motion optimization algorithm, which can help avoid excessive information exchange among nodes and conserve energy. TD-IMOCRP is proposed on the basis of IMOCRP to serve time-triggered traffic and event-driven traffic simultaneously, and special frame structure is designed to guarantee the priority of event-driven information delivery. In distributed clustering protocols, CRSNs nodes compare their CHs competition values with those of neighbors or random numbers to judge whether they can become CHs or not and then form clusters. Compared with centralized clustering protocols, distributed clustering protocols do not rely on the centralized processing capability of the sink and enable stronger network scalability. CogLEACH^11^, NSAC^12^ and WCM^13^ are representatives of distributed clustering protocols. Among them, CogLEACH calculates CHs competition value based on the number of idle channels perceived and compares it with a randomly generated number within [0,1] to determine CHs. NSAC sets CHs competition value according to node residual energy and available channels, and nodes with the highest competition values in the locality become CHs. WCM comprehensively calculates CHs competition value based on spatial correlation, sensing reputation and residual energy to determine CHs. The assumption of single-hop communication can simplify clustering routing protocol design. However, in actual large-scale CRSNs, the majority of nodes cannot reach the sink directly due to the restricted communication range, which will severely constrain the network surveillance capability.

### Multi-hop clustering routing protocols for CRSNs

Multi-hop clustering routing protocols for CRSNs are further categorized into uniform and uneven clustering protocols, and majority of them select CHs and form clusters in a distributed manner. In uniform clustering protocols, all CRSNs nodes exchange information within the same range (i.e., cluster radius) during CHs selection and cluster construction, such as DSAC^14^, EACRP^15^, SACR^16^, RFMCRP^17^ and EAQ-AODV^18^. In DSAC, each CRSNs node is clustered separately at the beginning, and then neighboring clusters are merged according to inter-cluster distance and channel information until the optimal number of clusters which is obtained by minimizing the total network energy consumption is achieved. In addition, inter-cluster routes are established to deliver data. EACRP also constructs clusters between the event and the sink by merging neighboring clusters, and CMs which have more residual energy, more available channels and are closer to the sink are chosen as gateways to assist in inter-cluster data forwarding. DSAC and EACRP facilitate strong scalability and stability, but excessive information exchange among nodes introduces huge control overhead. SACR takes dynamic spectrum availability and node energy consumption into consideration for proper CHs selection, and CHs rotation is introduced to further balance the residual energy among nodes. Relay nodes are determined based on cluster size, number of available channels and the Euclidean distance to the sink. RFMCRP is proposed based on nonlinear energy harvesting model, and energy level function-based selection criteria are defined to help select high-quality CHs and relays. Energy control mechanism is designed to manage the node status and help improve the cluster stability. EAQ-AODV determines CHs by leveraging energy-aware and Q learning-based reward mechanism, and relay nodes are chosen according to Ad hoc on-demand distance vector protocol to establish available routes. In this process, node residual energy, number of common available channels, number of hops, node communication range and trust factors are comprehensively taken into consideration. In uniform clustering protocols, CHs close to the sink are required to aggregate intra-cluster data and help forward data for outer layers, which will accelerate their energy exhaustion, result in energy holes and even network partition. To solve the above problems, uneven clustering protocols generally manage cluster radii to achieve effective inter-cluster energy balancing, in other words, the cluster radius of the CH close to the sink is reduced to decrease its energy consumption of receiving and aggregating intra-cluster data and reserve more energy for relaying packets for outer layers. LEAUCH^19^ is an uneven clustering protocol based on CogLEACH, and it calculates CHs competition value according to the number of idle channels. CRSNs nodes with CHs competition value larger than 0.4 become candidate CHs, and they will become final CHs if they possess the highest residual energy within cluster radius. LEAUCH calculates cluster radius for candidate CH *i* based on its Euclidean distance to the sink *d*_*i*,sink_ as below:

1 $$R_{c} = \left( {1 - c\frac{{d_{{{\text{max}}}} - d_{{i,{\text{sink}}}} }}{{d_{{{\text{max}}}} - d_{{{\text{min}}}} }}} \right)R_{c}^{0}$$

where *c* is a constant uneven clustering coefficient; *d*_max_ and *d*_min_ are the maximum and minimum Euclidean distance between all CRSNs nodes and the sink, respectively. $R_{c}^{0}$ is the maximum cluster radius of candidate CHs. However, the way of establishing inter-cluster routing is not provided, as a result, the performance of LEAUCH in multi-hop CRSNs cannot be validated. PSOEECA^20^ is an efficient channel assignment strategy based on particle swarm optimization (PSO) algorithm, and it leverages the similar way to calculate cluster radius, select CHs and form clusters. Node residual energy predicted by PSO algorithm is utilized to calculate fitness function, based on which channels are assigned for intra-cluster and inter-cluster communication. ESAUC^21^ is an energy and spectrum-aware uneven clustering protocol, and it further improves the cluster radius calculation of LEAUCH by taking the node residual energy, number of neighbors and channel idle probability into consideration as below:

2 $$R_{c} = \left[ {1 - \alpha \left( {\frac{{d_{{{\text{max}}}} - d_{{i,{\text{sink}}}} }}{{d_{{{\text{max}}}} - d_{{{\text{min}}}} }}} \right) - \beta \left( {1 - \frac{{E_{res} (i)}}{{E_{0} }}} \right) + \gamma \left( {1 - \frac{Neigh(i)}{{Neigh_{{{\text{max}}}} }}} \right) + \omega (1 - P_{a} )} \right]R_{c}^{0}$$

where *E*_*res*_(*i*) is the residual energy of node *i*; *E*_0_ is the initial energy of CRSNs nodes; *Neigh*(*i*) is the number of neighbors of node *i*; *Neigh*_max_ is the maximum number of neighbors of all CRSNs nodes; *P*_*a*_ is the channel idle probability;* α*, *β*, *γ* and *ω* are constant uneven clustering coefficients, but the value of *ω* is not provided. Therefore, cluster radius cannot be figured out, and the effectiveness of ESAUC cannot be tested.

The limitations of the existing multi-hop clustering routing protocols for CRSNs can be summarized as follows: (1) Research on extending the network lifetime mainly focuses on reducing the energy consumption of data delivery, but the huge energy consumption of control overhead is ignored; (2) When calculating cluster radius, uneven clustering coefficients are not adaptively configured according to specific network conditions. Instead, they are fixed; (3) Perfect spectrum sensing is generally assumed, i.e., there is no false alarm or missed detection during spectrum sensing. The above assumption can simplify clustering and routing, but it does not match the actual perceptual performance of CRSNs nodes. Actually, in order to protect PUs, CRSNs nodes should periodically perceive the occupancy states of licensed channels. Therefore, strong spectrum detection capability is vital for both PUs and CRSNs, to be specific, if false alarm occurs during spectrum detection, CRSNs nodes cannot leverage this channel for communication; if missed detection happens during spectrum detection and CRSNs nodes utilize this channel for communication, collisions with PUs will result in transmission failure. To conquer the above limitations, an imperfect spectrum sensing-based multi-hop uneven clustering routing protocol ISSMCRP is proposed in this paper. Cluster radii are adaptively configured, and control overhead and the corresponding energy consumption are strictly managed to conserve limited node battery energy.

## Design details of ISSMCRP protocol

**Figure 1:**
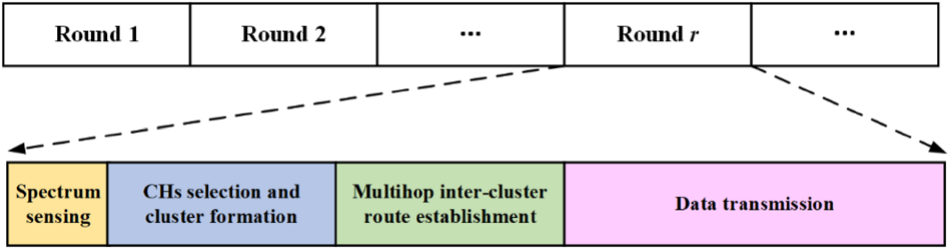
Round structure of ISSMCRP operation.

The operation of ISSMCRP is based on periodical rounds, and each round is divided into 4 stages, that is, spectrum sensing, CHs selection and cluster formation, multi-hop inter-cluster route establishment and data transmission, and the detailed timing diagram is shown in Fig. 1. The information obtained from spectrum sensing stage is leveraged to calculate the detection level function and idle detection accuracy of available channels, and they lay indispensable foundations for subsequent CHs selection and next-hop relay selection. The formed clusters and inter-cluster routes are used for data transmission.

### System model

In this paper, Semi-Markov model^22^ is leveraged to imitate dynamic occupancy behavior of PUs in the licensed spectrum. In this model, PU alternates between busy and idle states whose durations are independent random variables with parameters *P*_*busy*_ and *P*_*idle*_, respectively.

Assuming that the environmental noise is Gaussian white noise with mean 0 and variance of *δ*_*n*_^2^, and the signal from PU_*j*_ follows independent and identically distributed stochastic process with mean 0 and variance of *δ*_*x*,*j*_^2^. When energy detection-based method^23^ is applied for spectrum sensing, the false alarm probability and detection probability of node *i* (*i* = 1,2,…,*N*, *N* is the total number of CRSNs nodes) with respect to PU_*j*_ (*j* = 1,2,…,*M*, *M* is the total number of PUs randomly distributed in the network) are^24^:

3 $$P_{f(i,j)} = Q\left( {\frac{{\lambda - \delta_{n}^{2} }}{{\delta_{n}^{2} /\sqrt {Z/2} }}} \right)$$

4 $$P_{d(i,j)} = Q\left( {\frac{{\lambda - \left( {\delta_{n}^{2} + \delta_{x(i,j)}^{2} } \right)}}{{\left( {\delta_{n}^{2} + \delta_{x(i,j)}^{2} } \right)/\sqrt {Z/2} }}} \right)$$

where *Q*(•) is the Q function; *Z* is the number of samples and *Z* = *T*_*s*_ × *f*_*s*_, here, *T*_*s*_ is the spectrum sensing duration and *f*_*s*_ is the sampling frequency. *λ* is the energy detection threshold. According to Neyman-Pearson Lemma^25^, when *P*_*f*(*i*,*j*)_ is a small constant $\overline{P}_{f(i,j)}$, *λ* can be calculated according to Eq. (5). *δ*_*x*(*i*,*j*)_^2^ is the signal power received by node *i* from PU_*j*_, as shown in Eq. (6).

5 $$\lambda = \delta_{n}^{2} \times \left( {Q^{ - 1} \left( {\overline{P}_{f(i,j)} } \right)/\sqrt {Z/2} + 1} \right)$$

6 $$\delta_{x(i,j)}^{2} \left\{ \begin{gathered} = \left| {h_{(i,j)} } \right|\delta_{x,j}^{2} \, \quad \quad \,if \, d_{{to{\text{PU(}}i{,}j{)}}} \le {\text{IPR}} \hfill \\ \approx {0}\quad \quad \quad \quad \,otherwise \, \, \hfill \\ \end{gathered} \right.\,$$

where *d*_*to*PU(*i*,*j*)_ is the Euclidean distance from *i* to PU_*j*_. If *i* is within the interference protection range (IPR) of PU_*j*_, $\delta_{x(i,j)}^{2} = \left| {h_{(i,j)} } \right|\delta_{x,j}^{2}$, otherwise the received signal power can be approximated as 0. Here, *h*_(*i*,*j*)_ is the power gain between *i* and PU_*j*_, as shown in Eq. (7)^26^.

7 $$h_{{{(}i,j{)}}} = \left\{ {\begin{array}{*{20}c} {\frac{{G_{T} G_{R} l^{2} }}{{16\pi^{2} d_{{to{\text{PU}}(i,j)}}^{2} }}\quad \,\,if \,\, d_{{to{\text{PU(}}i,j{)}}} \le d_{0} } \\ {\frac{{G_{T} G_{R} h_{T}^{2} h_{R}^{2} }}{{d_{{to{\text{PU}}(i,j)}}^{4} }}\quad \quad otherwise \, } \\ \end{array} } \right.$$

where *G*_*T*_ and *G*_*R*_ are the gains of the transmission antenna and the receiving antenna, respectively; *l* is the wavelength of the transmission signal; *h*_*T*_ and *h*_*R*_ are the height of the transmission antenna and the receiving antenna, respectively. When *d*_*to*PU(*i*,*j*)_ ≤ *d*_0_, the signal attenuation follows free-space path loss, otherwise it follows multi-path loss. *d*_0_ is the distance threshold.

**Figure 2:**
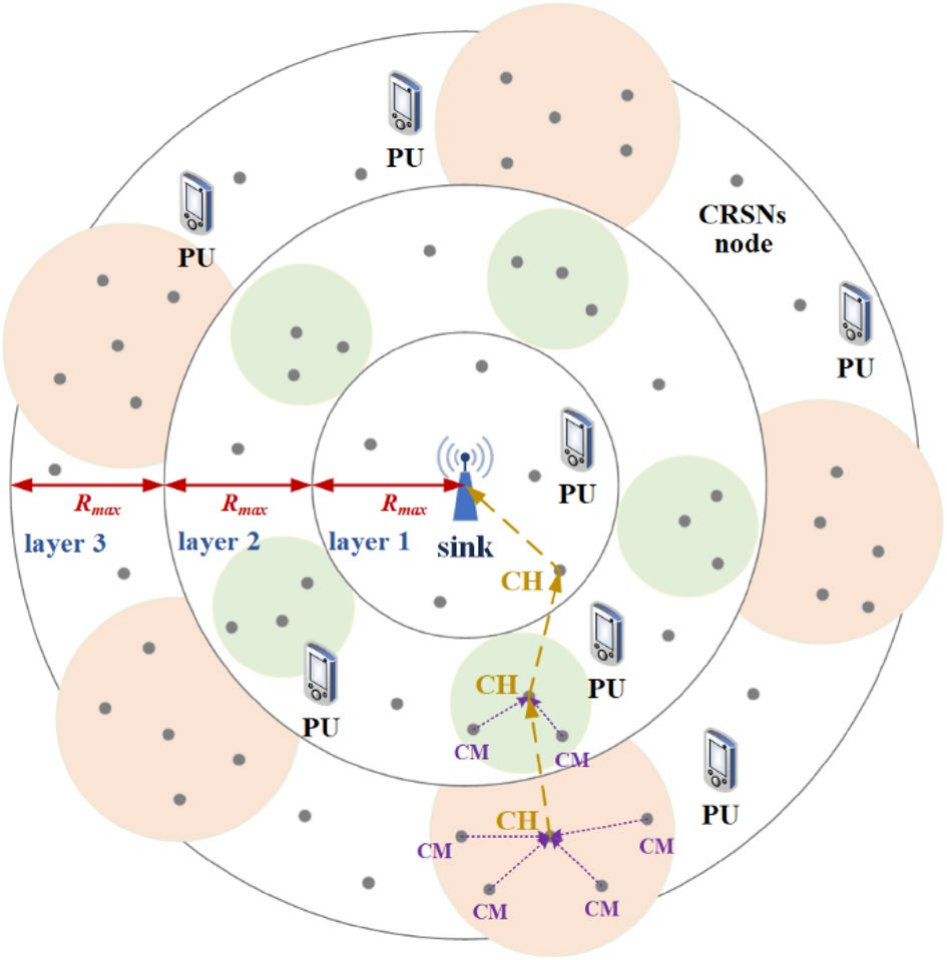
System architecture of cluster-based CRSNs.

According to the Euclidean distance between CRSNs nodes and the sink, nodes within the circular area with radius *R* are divided into different layers to limit the hop counts from CRSNs nodes to the sink and the corresponding energy consumption, and the width of each layer is set to *R*_*max*_, i.e., the maximum node transmission range. As shown in Fig. 2, the layer closest to the sink is layer 1, i.e., the layer number is a nondecreasing function of the Euclidean distance to the sink. CRSNs nodes update and store their own information and that of neighbors per round, and they automatically form clusters and search for multi-hop routes based on the above information. The detailed information stored by node *i* includes the following:

1. Its coordinate (*x*_*i*_,*y*_*i*_) and the Euclidean distance to the sink *d*_*i*,sink_, based on which it calculates its layer number *l*(*i*) as below:
  8 $$l\left( i \right) = \left\lceil {\frac{{d_{{i,{\text{sink}}}} }}{{R_{max} }}} \right\rceil$$
  where ⌈*x*⌉ denotes the minimum integer which is equal to or larger than *x*.
2. Its residual energy *E*_*res*_(*i*).
3. Set of available channels ***Channel***_***i***_ and number of available channels *C*(*i*).
4. Number of running neighbors *Neigh*(*i*). Here, neighbors of node *i* are defined as any node *j* which satisfies the following conditions: they are in the same layer, i.e., *l*(*i*) = *l*(*j*); *j* is within the circle centered at *i* and with radius of *R*_*c*_[*l*(*i*)]. In the worst case, node *i* may have no neighbors at all, but this will not affect its data transfer in most cases.
5. False alarm probability *P*_*f*(*i*,*t*)_ and detection probability *P*_*d*(*i*,*t*)_ with respect to each licensed channel *t* (*t* = 1,2,…,*C*, *C* is the total number of licensed channels).

As can be seen from Eqs. (3) and (4), when energy detection-based method is applied, the false alarm probability and detection probability of node *i* with respect to PU_*j*_ are *P*_*f*(*i*,*j*)_ and *P*_*d*(*i*,*j*)_, respectively. When there are multiple randomly distributed PUs and multiple licensed channels, the false alarm probability and detection probability of node *i* on channel *t* are:

9 $$P_{f(i,t)} = Q\left( {\frac{{\lambda - \delta_{n}^{2} }}{{\delta_{n}^{2} /\sqrt {Z/2} }}} \right)$$

10 $$P_{d(i,t)} = Q\left( {\frac{{\lambda - \left( {\delta_{n}^{2} + \sum\limits_{j = 1}^{M} {\delta_{x(i,j,t)}^{2} } } \right)}}{{\left( {\delta_{n}^{2} + \sum\limits_{j = 1}^{M} {\delta_{x(i,j,t)}^{2} } } \right)/\sqrt {Z/2} }}} \right)$$

where *δ*_*x*(*i*,*j*,*t*)_^2^ is the signal power received by *i* from PU_*j*_ on channel *t*, to be specific, if channel *t* is occupied by PU_*j*_, *δ*_*x*(*i*,*j*,*t*)_^2^ = *δ*_*x*(*i*,*j*)_^2^, otherwise node *i* cannot receive any signal power from PU_*j*_ on channel *t*, *δ*_*x*(*i*,*j*,*t*)_^2^ = 0.

6. Idle and occupancy probability of channel *t* under perfect spectrum detection, i.e., *P*_*off*(*i*,*t*)_ and *P*_*on*(*i*,*t*)_.

*P*_*Con*(*j*,*t*)_ and *P*_*Coff*(*j*,*t*)_ are the channel utilization ratio of *t* with respect to PU_*j*_ and the opposite ratio, respectively, and they can be calculated based on the historical channel occupancy statistics. CRSNs nodes cannot control the channel usage of PUs. Instead, they can only opportunistically access spectrum holes for communication. Therefore, when determining the idle and occupancy probability of each licensed channel, all PUs should be considered to adapt to various channel occupancy situations. When node *i* performs accurate spectrum sensing on channel *t*, if *i* is within the IPR of PU_*j*_, the probability that channel *t* is not occupied by PU_*j*_ and the opposite probability perceived by *i* are *P*_*Coff*(*j*,*t*)_ and *P*_*Con*(*j*,*t*)_, respectively; If *i* is out of the IPR of PU_*j*_, as the signal power received by *i* is so weak, it will judge that this channel is not occupied by PU_*j*_, i.e., *P*_*off*(*i*,*j*,*t*)_ = 1 and *P*_*on*(*i*,*j*,*t*)_ = 0. As stated above, *P*_*off*(*i*,*j*,*t*)_ and *P*_*on*(*i*,*j*,*t*)_ can be expressed as:

11 $$P_{{off{(}i,j,t{)}}} = \left\{ {\begin{array}{*{20}c} {P_{Coff(j,t)} \quad \,\,if \, d_{{to{\text{PU(}}i,j{)}}} \le {\text{IPR }}} \\ {1\quad \;\;\,\;\,\,\,\quad otherwise \, } \\ \end{array} } \right.$$

12 $$P_{{on{(}i,j,t{)}}} = \left\{ {\begin{array}{*{20}c} {P_{Con(j,t)} \quad \,\,if \, d_{{to{\text{PU(}}i,j{)}}} \le {\text{IPR }}} \\ {0\,\;\quad \,\,\,\,\,\,\,\,\;otherwise \, } \\ \end{array} } \right.$$

Under accurate spectrum detection, the idle and occupancy probability of channel *t* perceived by *i* are:

13 $$P_{{off{(}i,t{)}}} = \prod\limits_{j = 1}^{M} {P_{{off{(}i,j,t{)}}} }$$

14 $$P_{{on{(}i,t{)}}} = 1 - P_{{off{(}i,t{)}}}$$

Based on the false alarm and detection probability, idle and occupancy probability of licensed channels, *i* calculates its detection level function of available channels *P*_*CL*_(*i*) as below:

15 $$P_{CL} (i) = \sum\limits_{{t \in {{\varvec{Channel}}}_{\varvec{i}} }} {\left[ {P_{off(i,t)} \times (1 - P_{f(i,t)} ) - P_{on(i,t)} \times (1 - P_{d(i,t)} )} \right]}$$

where *t* is an idle channel perceived by node* i*, i.e., *t* ∈ ***Channel***_***i***_. *P*_*off*(*i*,*t*)_ × (1 − *P*_*f*(*i*,*t*)_) is the probability that channel *t* is actually idle and there is no false alarm during spectrum sensing; *P*_*on*(*i*,*t*)_ × (1 − *P*_*d*(*i*,*t*)_) is the probability that channel *t* is actually being occupied but there is missed detection during spectrum sensing. When channel *t* is perceived as idle, the missed detection probability which acts as penalty is subtracted from the correct detection probability. *P*_*CL*_(*i*) is the total detection level on all idle licensed channels perceived by *i*. Among them, idle channels with high sensing performance will make a great contribution to *P*_*CL*_(*i*), and channels with bad performance will contribute little to *P*_*CL*_(*i*). Therefore, using summation in Eq. (15) makes a compromise between the number of idle available channels and spectrum sensing performance.

If *t* ∈ ***Channel***_***i***_, the idle detection accuracy of channel *t* for *i* is:

16 $$P_{r(i,t)} = \frac{{P_{off(i,t)} \times (1 - P_{f(i,t)} )}}{{P_{off(i,t)} \times (1 - P_{f(i,t)} ) + P_{on(i,t)} \times (1 - P_{d(i,t)} )}}$$

where the denominator is the total probability that channel *t* is perceived as idle.

### CHs selection and cluster formation

Based on the detection level function of available channels *P*_*CL*_(*i*), *i* calculates its CHs competition value, as shown in Eq. (17). Nodes with stronger detection capability, higher residual energy and more neighbors are more likely to become CHs.

17 $$Compt(i) = P_{CL} (i) \times E_{res} (i) \times Neigh(i)$$

**Figure 3:**
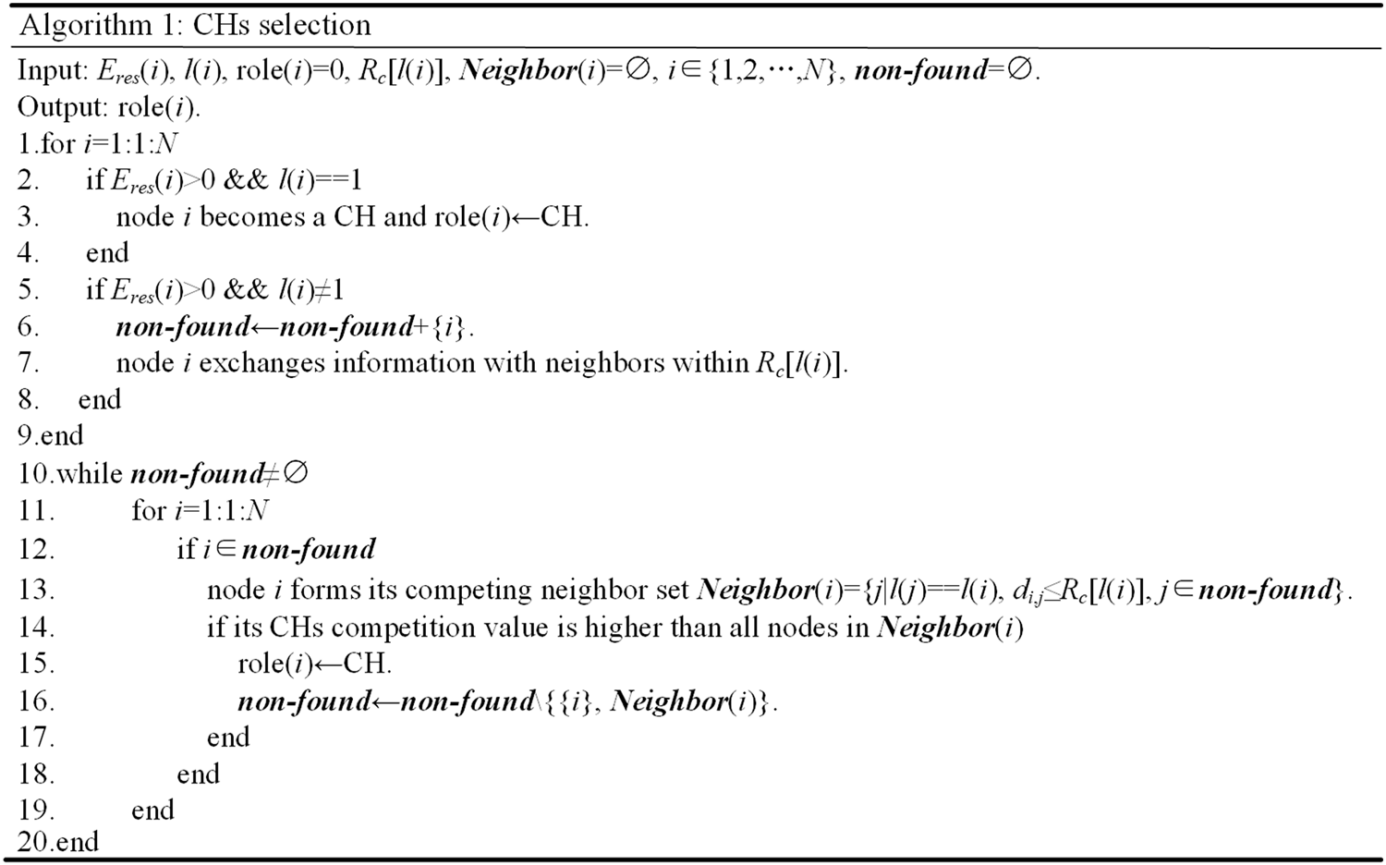
Pseudo code for CHs selection.

The pseudo code for CHs selection is shown in Fig. 3. Lines 2–4 show that all running nodes in layer 1 are clustered separately to eliminate the energy consumption caused by CHs selection and cluster formation. In this case, more energy is conserved to relay packets for other layers. As shown in lines 5–8, all running nodes except those in layer 1 broadcast their information and CHs competition values within cluster radius, and they also receive the corresponding information from neighbors within the same range. The cluster radius of *i R*_*c*_[*l*(*i*)] is calculated by:

18 $$\frac{{R_{c} [l(i)]}}{{R_{0} }} = \frac{{\rho \pi \left[ {l^{2} (i) - [l(i) - 1]^{2} } \right]R_{max}^{2} }}{{\rho \pi \left[ {\left\lceil {\frac{R}{{R_{max} }}} \right\rceil^{2} - \left( {\left\lceil {\frac{R}{{R_{max} }}} \right\rceil - 1} \right)^{2} } \right]R_{max}^{2} }}$$

where *ρ* is the node density, and *R*_0_ is the range within which CHs selection and cluster formation in the outermost layer takes place. In this paper, the ratio of *R*_*c*_[*l*(*i*)] and *R*_0_ is set to the ratio of the number of nodes in layer *l*(*i*) and the outermost layer to balance the CHs distribution among different layers. *R*_0_ is set as *R*_*max*_, correspondingly, *R*_*c*_[*l*(*i*)] can be simplified into:

19 $$R_{c} [l(i)] = \frac{{l^{2} (i) - [l(i) - 1]^{2} }}{{\left\lceil {\frac{R}{{R_{max} }}} \right\rceil^{2} - \left( {\left\lceil {\frac{R}{{R_{max} }}} \right\rceil - 1} \right)^{2} }} \times R_{max}$$

The function of setting reasonable cluster radii can be analyzed from the following 2 aspects: (1) The total energy consumption of control information exchange is reduced. On the one hand, the range of broadcasting control information is restricted within cluster radius instead of *R*_*max*_. As a result, the corresponding energy consumption is decreased, as it is positively proportional to the broadcasting range; On the other hand, neighbors are defined as the nodes within cluster radius instead of within *R*_*max*_, and the decrease of the number of neighbors results in fewer received control packets. Consequently, the energy consumption of control information reception declines. (2) The number of intra-cluster members is decreased, and the energy consumption of receiving and aggregating intra-cluster data at CHs is also reduced.

Lines 10–20 represent the CHs competition in the locality. If the CHs competition value of a node is smaller than one of its neighbors, it will quit from competition, otherwise it will become a CH. The above process is repeated until all nodes become CHs or quit from competition.

**Figure 4:**
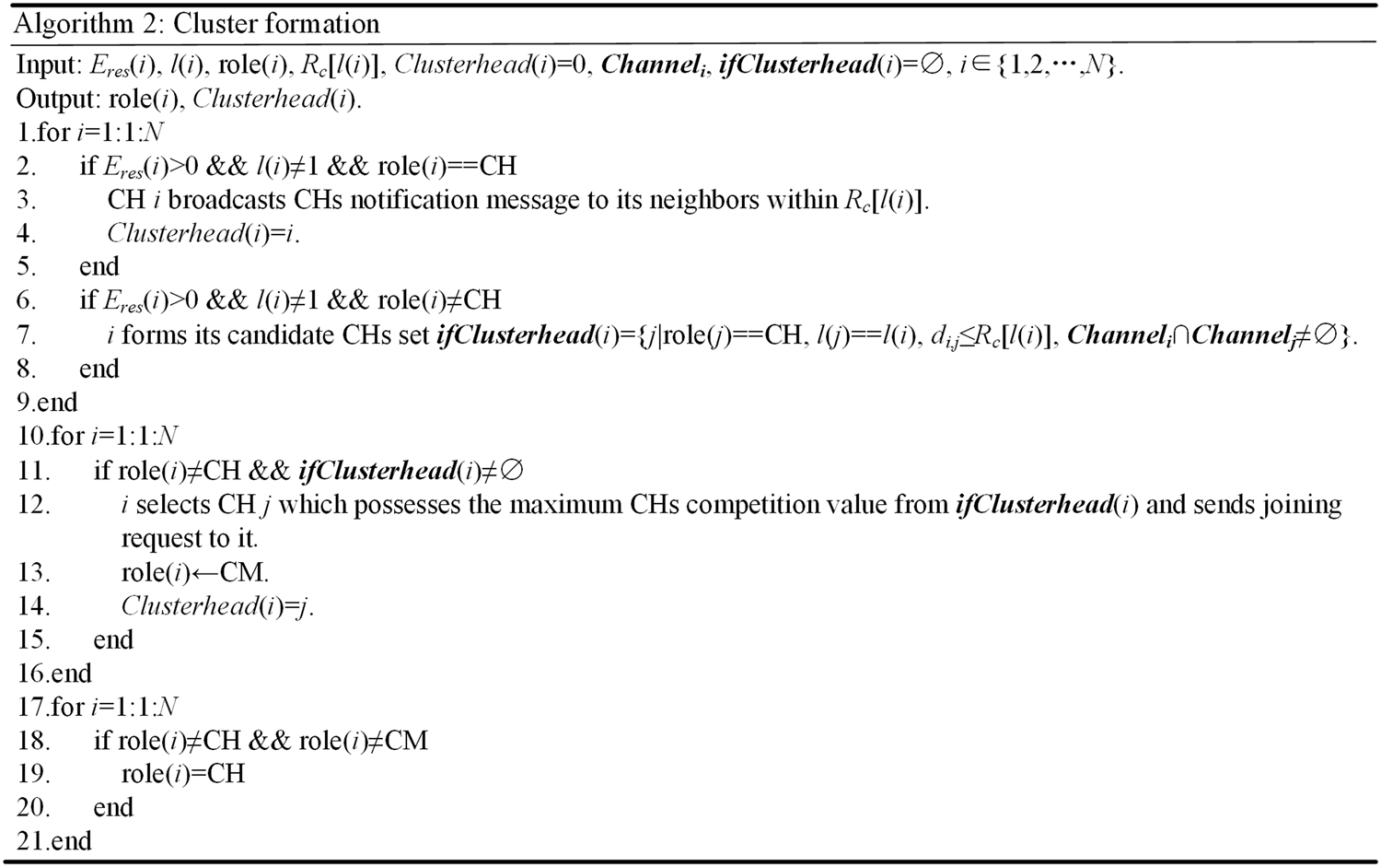
Pseudo code for cluster formation.

The pseudo code for cluster formation is shown in Fig. 4. Lines 2–5 denote that all CHs except those in layer 1 broadcast CHs notification message within cluster radius. NonCHs nodes join clusters by sending out joining request to the CHs which possess the highest CHs competition values and share common available channels, and corresponding CHs receive the request and store them into CMs list, as shown by lines 6–16. Lines 17–21 show that nodes which cannot receive any CHs notification message become independent CHs.

**Figure 5:**
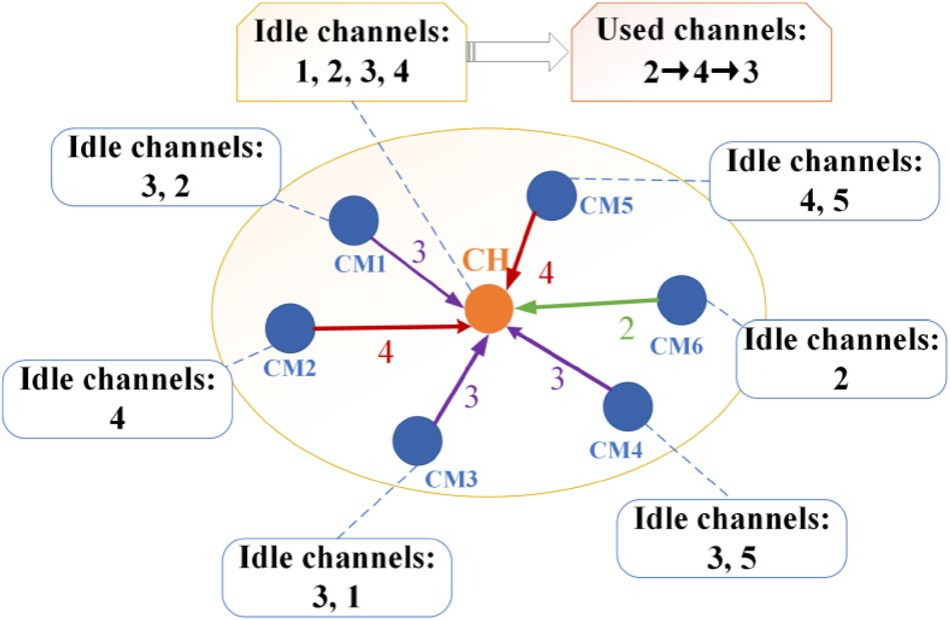
Cluster channel selection and channel switching.

During cluster channel selection, in order to promote successful intra-cluster packet delivery, idle channels should be accurately chosen to perform data transmission. CMs calculate idle detection accuracy of each available channel according to Eq. (16), and the channel which is also available at the CH and possesses the highest *P*_*r*(*i*,*t*)_ is determined as cluster channel. As CMs may select different cluster channels, the CH should leverage channel switching to aggregate intra-cluster data. In order to reduce channel switching as much as possible, the CH will schedule the data transmission from its CMs on the same channel successively, and then it will switch to another channel until all CMs complete their data transmission. Figure 5 exhibits how ISSMCRP selects cluster channels and performs channel switching. To be specific, the numbers besides each node represent its available channels, for example, channels 1, 2, 3 and 4 are idle at the CH. CMs rank their available channels in descending order of *P*_*r*(*i*,*t*)_. For example, CM1 ranks its available channels and the sequence is channel 3 first and then channel 2. 6 CMs choose their own cluster channels which are shared by their CH and possess the highest *P*_*r*(*i*,*t*)_ value. CM1, CM3 and CM4 choose channel 3; CM2 and CM5 select channel 4, while CM6 determines channel 2 as its cluster channel. The CH exploits channel switching to gather intra-cluster data, and the channel switching sequence is determined by ascending order of number of CMs which select corresponding cluster channels. Firstly, the CH receives data from CM 6 on channel 2; Secondly, the CH switches to channel 4 to gather information from CM2 and CM5; Finally, the CH accesses channel 3 to receive data from CM1, CM3 and CM4. Through the above cluster channel selection and channel switching, the CH aggregates all data from its CMs.

### Multi-hop inter-cluster route establishment

Nodes in layer 1 can reach the sink through single hop, and they send their data to the sink directly. Other CHs need to select proper relays to help forward their data until it reaches the sink. The relay competition value of node *i* is defined as:

20 $$Relaycompt\left( i \right) = P_{CL} \left( i \right) \times E_{res} (i) \times \frac{1}{{E_{elec} + E_{fs} \times d_{{i,{\text{sink}}}}^{2} }}$$

where *E*_*elec*_ is the energy consumption of transceiver electronics per bit, and *E*_*fs*_ is the energy consumption coefficient of power amplifier. The denominator is the energy consumption of forwarding 1 bit of data towards the sink. Nodes with stronger spectrum sensing capability, higher residual energy and closer to the sink have higher probability of being next-hop relays. If the selected relay is in layer 1, the route establishment ends up, otherwise the next-hop relay selection continues.

**Figure 6:**
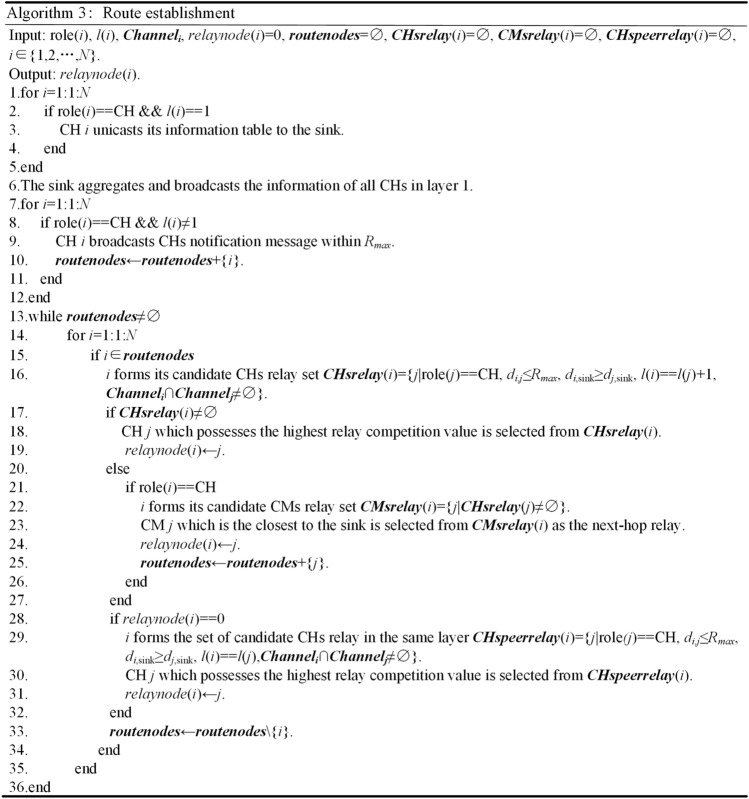
Pseudo code for route establishment.

The pseudo code for route establishment is shown in Fig. 6. Lines 1–6 show that CHs in layer 1 directly send their information to the sink which will aggregate and broadcast the received information to nodes in layer 2. In this case, the energy consumption of broadcasting information (for nodes in layer 1) and that of receiving the corresponding information (for nodes in layer 2) are both reduced. In lines 7–12, nonlayer 1 CHs broadcast CHs notification message within *R*_*max*_. CHs in the same layer and neighbors in the next outer layer receive the information which can be used for next-hop relay selection. Lines 16–32 show how to select next hops. The CH in the next inner layer is preferred, i.e., the CH in the next inner layer which is within the communication range, shares common idle channels and closer to the sink is selected. If such CH cannot be found, the CM which is closest to the sink and can communicate with a CH in the next inner layer is selected, as shown in lines 21–26. If such next hop cannot be found either, the CH which is in the same layer, shares common available channels, possesses higher relay competition value and closer to the sink will be selected, as shown in lines 28–32. The above process is repeated until relays in layer 1 are found or no available route can be found.

As all relays have obtained information about their next hops during control information exchange, they calculate the idle detection accuracy of each common available channel according to Eq. (16), and the one with the highest *P*_*r*(*i*,*t*)_ is determined as inter-cluster channel to improve inter-cluster packet delivery ratio.

After cluster formation and route establishment, CHs allocate time slots to schedule the data transmission from their CMs, and CMs transmit data towards their CHs in the assigned time slots on cluster channels. CHs forward the aggregated data towards the sink through inter-cluster data relaying.

## Results and discussion

Matlab is utilized to implement ISSMCRP, and its advantages of extending the network lifetime and enhancing the network surveillance capability are verified through performance comparisons with the existing clustering routing protocols for CRSNs, such as CogLEACH^11^, NSAC^12^, WCM^13^ and DSAC^14^. 450 homogeneous CRSNs nodes and 50 PUs are randomly and uniformly distributed in the circular area with radius 150 m, and the sink is located at the center^9,17,19,27^. Other parameter settings are shown in Table 2.

**Table 2:** Simulation parameter settings.

Parameters	Values
Busy probability of PUs *P*_*busy*_	0.8
Idle probability of PUs *P*_*idle*_	0.2
Noise power $\delta_{n}^{2}$	0.00004W
Variation of PUs signal $\delta_{x}^{2}$	1W
Total number of CRSNs nodes *N*	450
Total number of PUs *M*	50
Number of samples in spectrum sensing *Z*	1000
Constant $\overline{P}_{f}$	0.01
Interference protection range of PUs IPR	20 m
Distance threshold* d*_0_	87.7 m
Network radius* R*	150 m
The maximum communication range of CRSNs nodes *R*_*max*_	50 m
Total number of licensed channels* C*	5
Energy consumption of transceiver electronics per bit *E*_*elec*_	50nJ/bit
Energy consumption coefficient of power amplifier *E*_*fs*_	10pJ/bit/m^2^

### Network lifetime comparison

**Figure 7:**
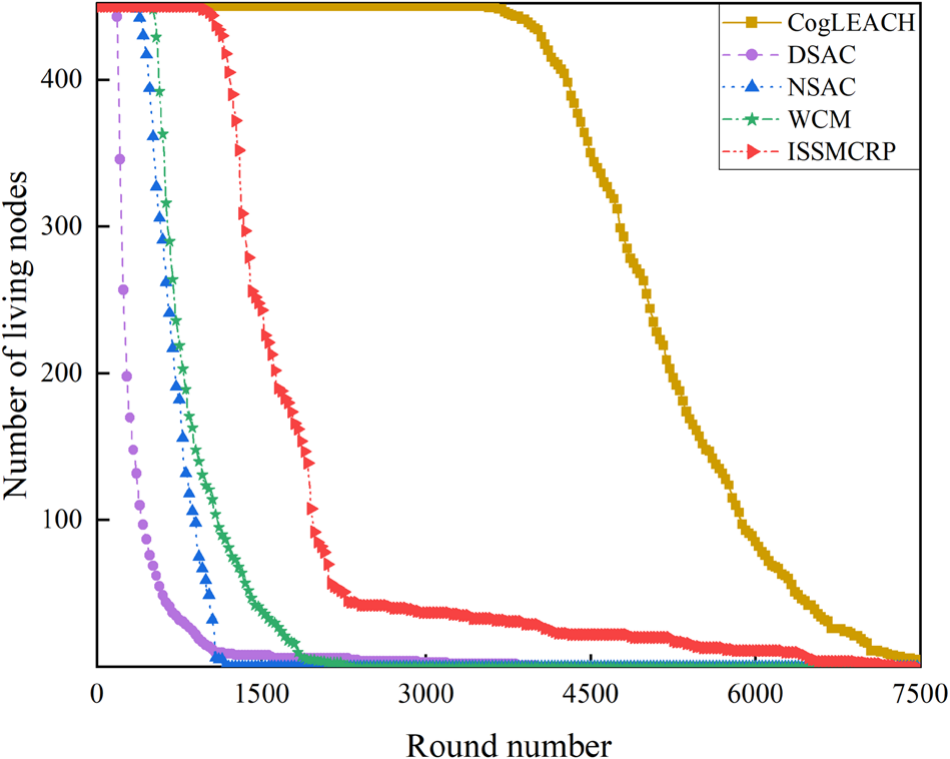
Comparison results of number of living nodes.

Each CRSNs node consumes energy for information transmission and reception. As *R*_*max*_ ≤ *d*_0_, free-space path loss model is leveraged to quantify the corresponding energy consumption and lays indispensible foundations for performance analysis. More details can be found in^27^. Network lifetime is one of the most important metrics for evaluating the performance of clustering routing protocols for CRSNs, and it can be analyzed from number of living nodes per round, as shown in Fig. 7.

As can be seen from Fig. 7, ISSMCRP achieves much better performance than DSAC, NSAC and WCM but worse than CogLEACH in terms of the number of living nodes. In order to verify this, the first node death time and the last node death time (in round) are recorded, and the results are shown in Table 3.

**Table 3:** Comparison results of network lifetime.

Protocols	The first node death time (in round)	The last node death time (in round)
CogLEACH	3546	7912
DSAC	166	3997
NSAC	340	1146
WCM	504	2282
ISSMCRP	949	7273

**Figure 8:**
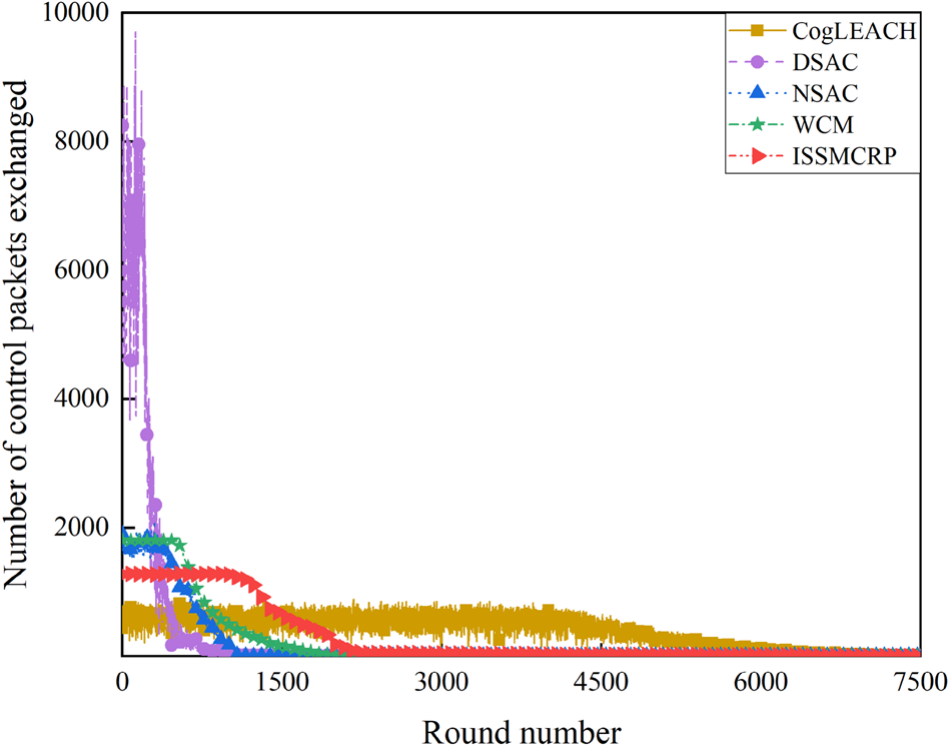
Comparison results of control overhead.

**Figure 9:**
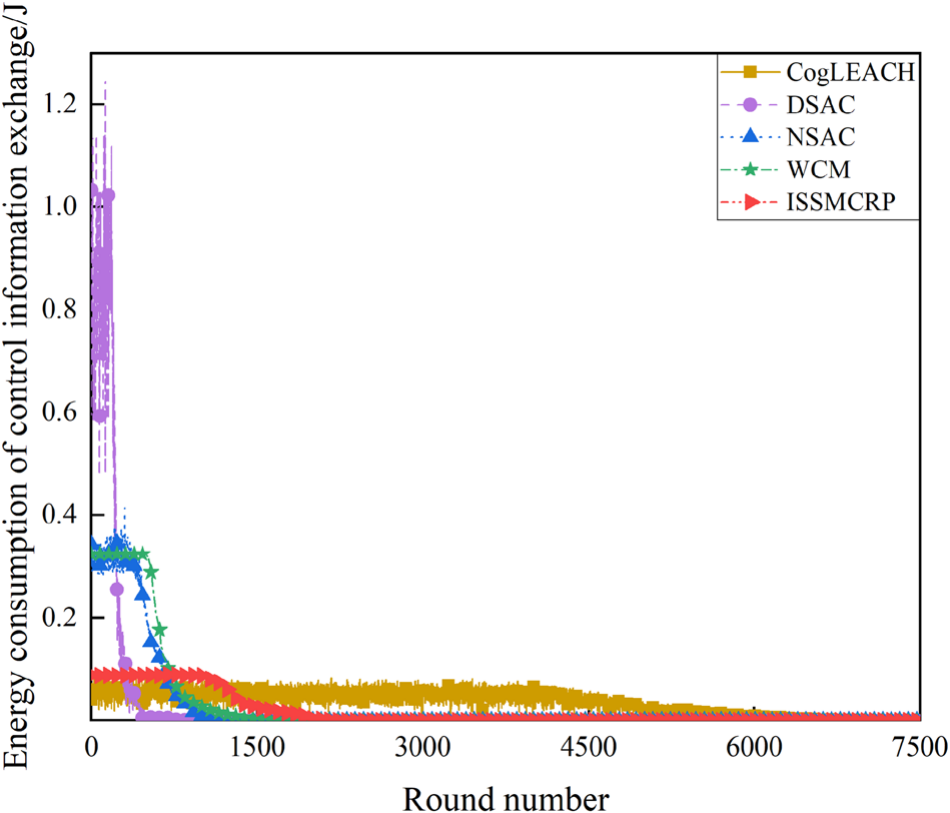
Comparison results of energy consumption of control information exchange.

**Figure 10:**
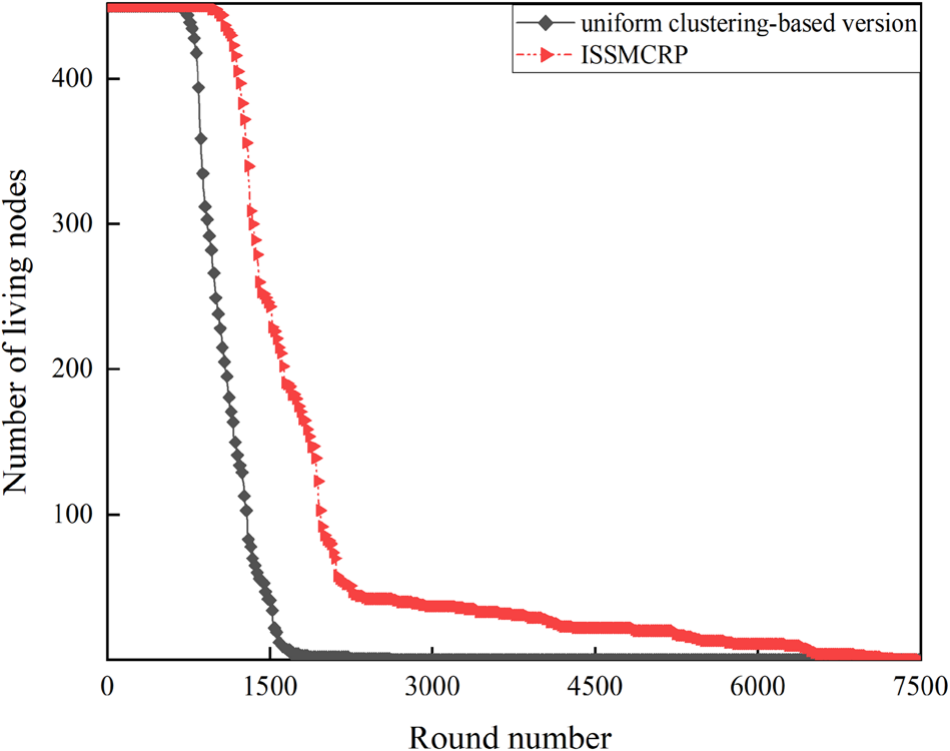
Benefit verification of uneven clustering in ISSMCRP.

As shown in Table 3, for ISSMCRP, the first death node appears in round 949 and the last death node appears in round 7273, which are both later than that of DSAC, NSAC and WCM but earlier than CogLEACH. It indicates that ISSMCRP gains obvious advantages in reducing node energy consumption and prolonging the network lifetime over other competing protocols except CogLEACH. The reasons are analyzed as follows: (1) ISSMCRP strictly limits the control overhead and the corresponding energy consumption introduced by CHs selection and cluster formation to conserve node energy. To be specific, all running nodes in layer 1 are clustered separately, which eliminates the control overhead of CHs selection and cluster formation. All other running nodes broadcast their information and CHs competition values within cluster radius; CHs broadcast CHs notification messages; NonCHs nodes broadcast quit messages, and they join certain clusters by sending out joining requests. Therefore, the control overhead of CHs selection and cluster formation in ISSMCRP is less than 3 times the number of living nodes. In addition, ISSMCRP manages the range of control information exchange, i.e., it only allows CRSNs nodes to exchange control information within cluster radius. As the cluster radii of all layers except the outmost layer are much smaller than *R*_*max*_, the energy consumption of control information exchange is reduced. In order to show the impact of control information exchange on the network lifetime, the number of control packets exchanged and the corresponding energy consumption are shown in Figs. 8 and 9, respectively. To achieve the optimal number of clusters obtained from theoretical derivation, DSAC requires extensive information exchange among nodes to judge whether merging conditions between neighboring clusters are satisfied. NSAC requires all nonclustered nodes constantly update and broadcast CHs weights, and nodes with the highest weight values become CHs. The above process is repeated until all nodes are clustered. Correspondingly, the control overhead of DSAC and NSAC during CHs selection and cluster formation is pretty high. The control overhead of WCM during CHs selection and cluster formation is about 4 times the number of living nodes, which is higher than ISSMCRP. To be specific, all nodes broadcast spectrum sensing information and CHs weight; CHs broadcast CHs notification messages; NnonCHs nodes broadcast quit messages, and they also send out joining requests; CHs transmit cluster information towards the sink. The number of control packets exchanged during CHs selection and cluster formation in CogLEACH is about twice the number of living nodes, that is, CHs broadcast temporary and final CHs notification messages while nonCHs nodes send out temporary joining requests and final confirmation messages. As analyzed above, the control overhead of CogLEACH is lower than ISSMCRP, which is consistent with Fig. 8. However, as can be seen from Fig. 9, the gap between CogLEACH and ISSMCRP is smaller than that in Fig. 8. This is because the range of control information exchange in CogLEACH is set as *R*_*max*_, and larger range means more energy consumption. This just demonstrates the necessity of setting cluster radii. Although the energy consumption of control information exchange of ISSMCPR is comparable with or even less than CogLEACH, its energy consumption of data transmission is much higher, as it delivers many more packets. This will be analyzed from the network surveillance capability aspect, and more details can be found in later parts of this paper. (2) ISSMCRP forms uneven clusters throughout the whole network by controlling cluster radii, and it enables inner CHs to conserve energy by decreasing the energy consumption of receiving and aggregating intra-cluster data, which can help balance the residual energy among nodes and prolong the network lifetime. In order to test the benefits brought by uneven clustering, ISSMCRP is compared with its peer version which applies uniform clustering (cluster radii of all layers are set to *R*_*max*_), and the results are shown in Fig. 10.

As shown in Fig. 10, the first death node appears in round 682 and the last death node appears in round 2608 in the uniform clustering-based version of ISSMCRP, which are both obviously earlier than ISSMCRP. It demonstrates that uneven clustering helps ISSMCRP dramatically extend the network lifespan.

### Network surveillance capability comparison

**Figure 11:**
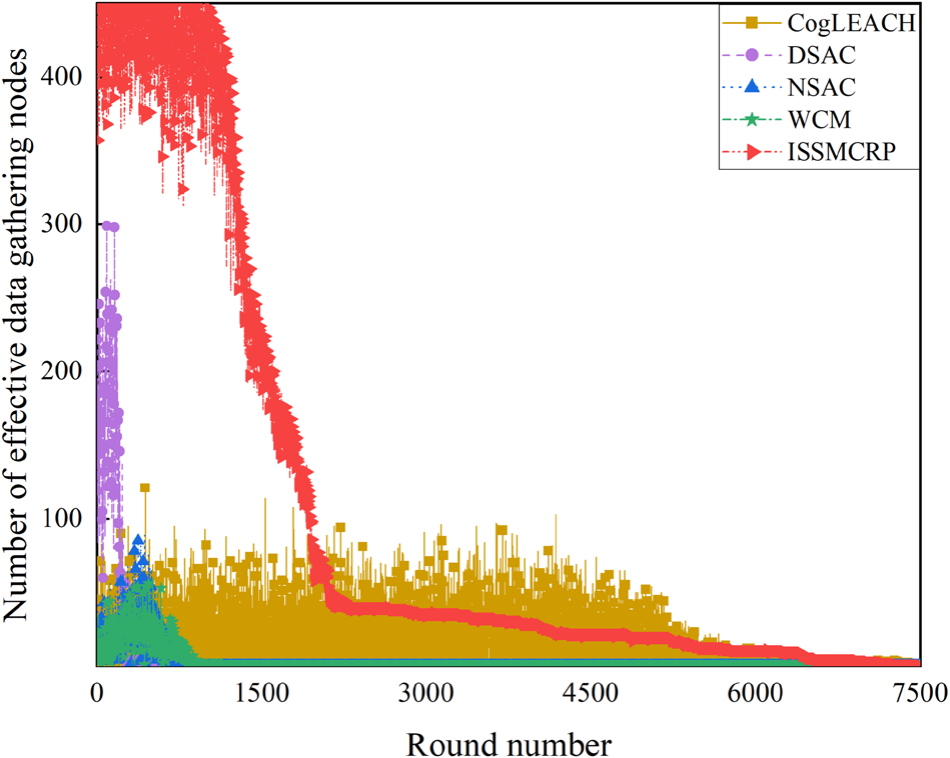
Comparison results of number of effective data gathering nodes.

Network surveillance capability is also an important performance metric of clustering routing protocols for CRSNs, and it can be observed from number of effective data gathering nodes per round, as shown in Fig. 11.

**Figure 12:**
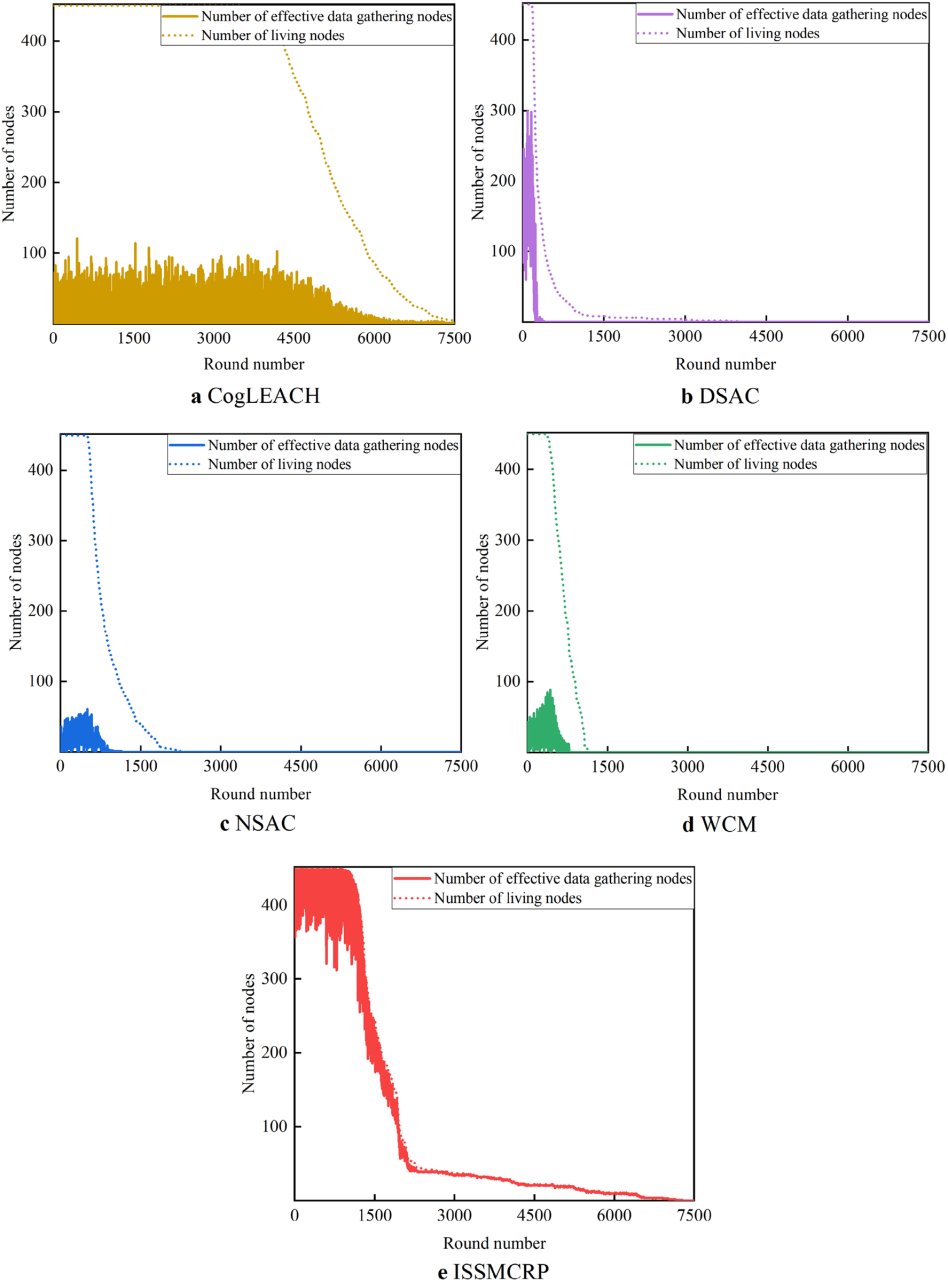
Comparison of number of effective data gathering nodes and number of living nodes.

**Figure 13:**
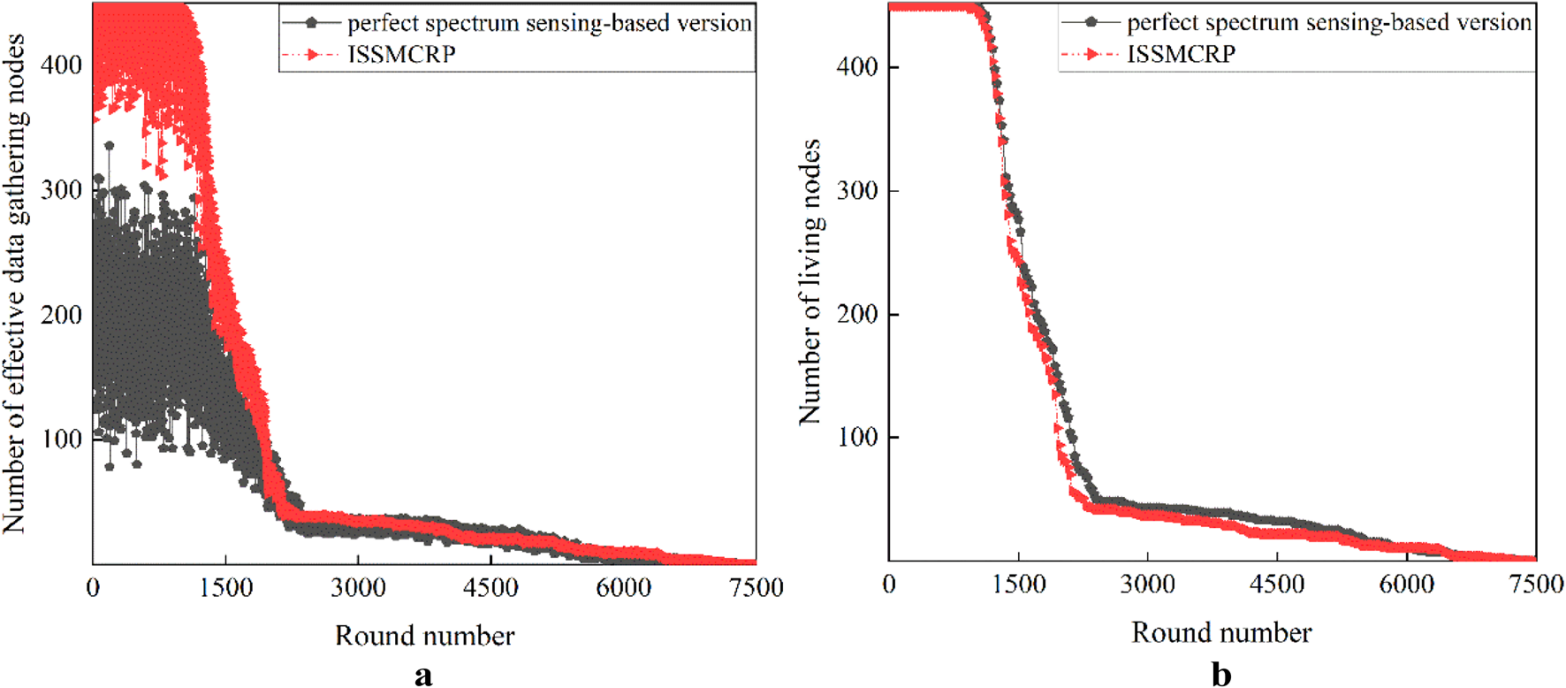
The necessity of taking the impact of imperfect spectrum sensing into consideration.

From Fig. 11, we can observe that the number of effective data gathering nodes in ISSMCRP is much higher than DSAC, NSAC and WCM. In addition, before round 2095, the number of effective data gathering nodes in ISSMCRP is also obviously higher than CogLEACH, while they approximate with each other after round 2095. As can be seen from Fig. 7, there are many more living nodes in CogLEACH after round 2095, which means that only a small fraction of nodes in CogLEACH can effectively collect data. In order to further analyze their information collection capability, the number of effective data gathering nodes and number of living nodes are compared, and the results are shown in Fig. 12. There is a big difference between the two curves in CogLEACH, DSAC, NSAC and WCM, while the tendency of the two curves in ISSMCRP is almost the same, which indicates that ISSMCRP can guarantee effective data collection and improve the network surveillance capability. The reasons are listed below: (1) CogLEACH, NSAC and WCM are all single-hop clustering routing protocols for CRSNs, and they consider nothing about inter-cluster routing. As a result, only the CHs which can reach the sink directly can achieve successful data delivery, which heavily restricts the network surveillance capability. DSAC and ISSMCRP are multi-hop clustering routing protocols, and majority of nodes can transmit their data packets to the sink through data relaying. Therefore, there are more effective data gathering nodes in DSAC and ISSMCRP. (2) Imperfect spectrum sensing i.e., false alarm and missed detection may occur during spectrum sensing stage. To be specific, if there is false alarm, nodes cannot utilize the available channels for communication; if there is missed detection and this channel is chosen for communication, collisions with PUs will occur, which will lead to transmission failure. CogLEACH, DSAC, NSAC and WCM all leave imperfect spectrum sensing out of consideration, while its impact has always been taken into consideration during CHs selection, cluster formation and route establishment in ISSMCRP. In order to validate the necessity of considering imperfect spectrum sensing, ISSMCRP is compared with its peer version which assumes perfect spectrum sensing, and the results are shown in Fig. 13.

As shown in Fig. 13a, before round 1942, the number of effective data gathering nodes in ISSMCRP is obviously higher than its counterpart, but its number of living nodes is a little lower, as shown in Fig. 13b. In other words, ISSMCRP guarantees effective network surveillance at the cost of increasing node energy consumption to a certain extent. After round 1942, although they achieve similar number of effective data gathering nodes, the data gathering capability of ISSMCRP is more powerful. For quantitative comparison, the average packet delivery ratio is calculated, and it is defined as the ratio between the total number of effective data gathering nodes in all rounds and the number of living nodes. The average packet delivery ratio of the perfect spectrum sensing-based version is 0.4836, and it is much lower than ISSMCRP whose value is 0.9231. The necessity of considering imperfect spectrum sensing is verified. The average packet delivery ratio of other protocols is given in Table 4.

**Table 4:** Comparison results of average packet delivery ratio.

Protocols	Average packet delivery ratio
CogLEACH	0.0484
DSAC	0.2234
NSAC	0.0524
WCM	0.0433
ISSMCRP	0.9231

As stated above, compared with DSAC, NSAC and WCM, ISSMCRP reduces the control overhead introduced by CHs selection and cluster formation, and it also leverages uneven clustering to balance the residual energy among CHs, which can significantly extend the network lifespan. Compared with CogLEACH, ISSMCRP establishes inter-cluster routing and always takes imperfect spectrum sensing into account, which helps improve the network surveillance capability. In a word, ISSMCRP possesses the dual advantages of expanding the network lifespan and enhancing the network surveillance capability.

## Conclusions

Current clustering routing protocols designed for CRSNs usually assume perfect spectrum sensing, which does not match the actual perceptual performance of CRSNs nodes. To achieve effective data delivery, an imperfect spectrum sensing-based multi-hop clustering routing protocol ISSMCRP is proposed for CRSNs in this paper. Detection level function and idle detection accuracy of available channels are leveraged to quantify the impact of imperfect spectrum sensing. On this basis, CHs and relays with high spectrum sensing capability are chosen, and accurate idle channels are preferred to transmit data. Simulation results show that ISSMCRP gains obvious advantages in prolonging the network lifetime and improving the network surveillance capability. To be specific, compared with DSAC, NSAC and WCM, the network lifetime is extended by over 88 percent; Compared with CogLEACH, the average packet delivery ratio is enhanced by more than 87 percent. However, ISSMCRP cannot provide energy supplement for CRSNs nodes, and when their residual energy is exhausted, they cannot function any more. Therefore, in our future work, we plan to exploit energy harvesting and simultaneous wireless information and power transfer technique to compensate and balance the node residual energy to further expand the network lifespan.

## Data Availability

The datasets generated during and/or analysed during the current study are available from the corresponding author on reasonable request.
